# Light-Weight Student LSTM for Real-Time Wildfire Smoke Detection

**DOI:** 10.3390/s20195508

**Published:** 2020-09-25

**Authors:** Mira Jeong, MinJi Park, Jaeyeal Nam, Byoung Chul Ko

**Affiliations:** Department of Computer Engineering, Keimyung University, Daegu 42601, Korea; mystroll@kmu.ac.kr (M.J.); anmj8780@stu.kmu.ac.kr (M.P.); jynam@kmu.ac.kr (J.N.)

**Keywords:** wildfire smoke, YOLOv3, LSTM, teacher-student framework, smoke-tube, student LSTM

## Abstract

As the need for wildfire detection increases, research on wildfire smoke detection combining low-cost cameras and deep learning technology is increasing. Camera-based wildfire smoke detection is inexpensive, allowing for a quick detection, and allows a smoke to be checked by the naked eye. However, because a surveillance system must rely only on visual characteristics, it often erroneously detects fog and clouds as smoke. In this study, a combination of a You-Only-Look-Once detector and a long short-term memory (LSTM) classifier is applied to improve the performance of wildfire smoke detection by reflecting on the spatial and temporal characteristics of wildfire smoke. However, because it is necessary to lighten the heavy LSTM model for real-time smoke detection, in this paper, we propose a new method for applying the teacher–student framework to deep LSTM. Through this method, a shallow student LSTM is designed to reduce the number of layers and cells constituting the LSTM model while maintaining the original deep LSTM performance. As the experimental results indicate, our proposed method achieves up to an 8.4-fold decrease in the number of parameters and a faster processing time than the teacher LSTM while maintaining a similar detection performance as deep LSTM using several state-of-the-art methods on a wildfire benchmark dataset.

## 1. Introduction

Among the various natural disasters caused by global warming, wildfires have a higher risk than other natural disasters and result in greater damage to property, human life, and ecosystems. A representative example is a wildfire that occurred in the Krasnoyarsk region of Siberia, Russia in the summer of July 2020, and wildfires in California, USA, in August of the same year. The most serious of recent wildfires were those in Queensland, Australia in June 2019, which lasted for approximately 6 months, and burned down approximately 5900 buildings and over 18.6 million hectares of land, killing at least 34 people [1]. According to the report of National Interagency Fire Centre of USA [2], over the past 10 years, there were an average of 64,100 wildfires annually and an average of 6.8 million acres burned annually in USA.

Unlike natural disasters such as earthquakes and tsunamis, wildfires are often caused by human carelessness rather than natural occurrences, and the amount of damage can be large; however, they are also detectable earlier than other disasters. Therefore, if a wildfire can be detected early, the damage to people and property can be minimised.

The most basic way to monitor wildfires is to install a watchtower on top of a high mountain and watch their development with the naked eye. However, it is impossible for a person to monitor extensive wildfires throughout the day. To solve this problem, studies on an automated wildfire monitoring system using sensor-based Internet-of-Things (IoT) technology have recently been conducted. Sensor-based wildfire detectors mostly use wireless sensor networks (WSNs), which are composed of interconnected sensors installed in a dense configuration. WSN sensor nodes collect measurement data such as the relative humidity, temperature, smoke, sound, and wind speed, all of which are required for determining the degree of danger of the wildfires [3,4]. With wildfire detection using a WSN [5], the exact location and range of spread of a fire can be predicted; however, the sensors need to be installed in a large area, and if some sensors malfunction or break down, the overall network prediction performance can be reduced. In addition, when a fire alarm is triggered by a sensor, the manager has the problem of receiving additional information such as whether the wildfire has actually occurred, the direction of the spread, the size, and the on-site characteristics of the wildfire. For these reasons, sensors are more suitable for monitoring indoor fires than outdoor wildfires.

The most efficient device in terms of accuracy and cost in detecting wildfires is the use of a camera. Camera-based wildfire detection can be divided into static and dynamic methods. Using a dynamic approach, unmanned aerial vehicles (UAVs) with a camera and global positioning system (GPS) or infrared (IR) sensors [6,7,8] installed are widely used in wildfire detection because of their easy movement and flight capability. Although wildfire detection using UAVs has the advantage of being able to monitor a wider area than a WSN-based method, and knowing the current state and direction of the spread, the operating time is short and the search range is limited owing to an insufficient battery capacity and the distance limitations of a wireless network. Although satellites [9,10] can be used to detect fires within a wide area, they have a disadvantage in that it is difficult to detect fires during the initial stage, and the time during which a fire can be detected is limited depending on the revisiting cycle of the satellite.

The most commonly used method is to monitor wildfires using only static cameras installed on the surveillance tower of a mountain [11,12,13]. In this system, camera sensors are installed at top of a mountain and transfer image sequences to a monitoring server using wired or wireless network. The transmitted image sequences are analysed by a wildfire detection system in real-time, and the system automatically monitors whether wildfire is possible. If an emergency situation is detected, the warning system sounds an alarm and sends image sequences of the remote site to the control centre for a manager’s visual check of whether it is an actual emergency or not [14]. As the biggest advantage of wildfire detection using a camera, when a wildfire alarm occurs, the manager can visually check whether an actual fire has been detected through the video camera placed on site without the need to visit the location. As another advantage, a camera-based wildfire detection method can be used to search a wide area in real time at low cost while showing a performance similar to that of other sensors. In addition, unlike using UAVs and satellites, camera-based fire detectors can operate 24 h a day. Therefore, in this paper, we focus on a wildfire detection system using a static camera.

**Figure 1:**
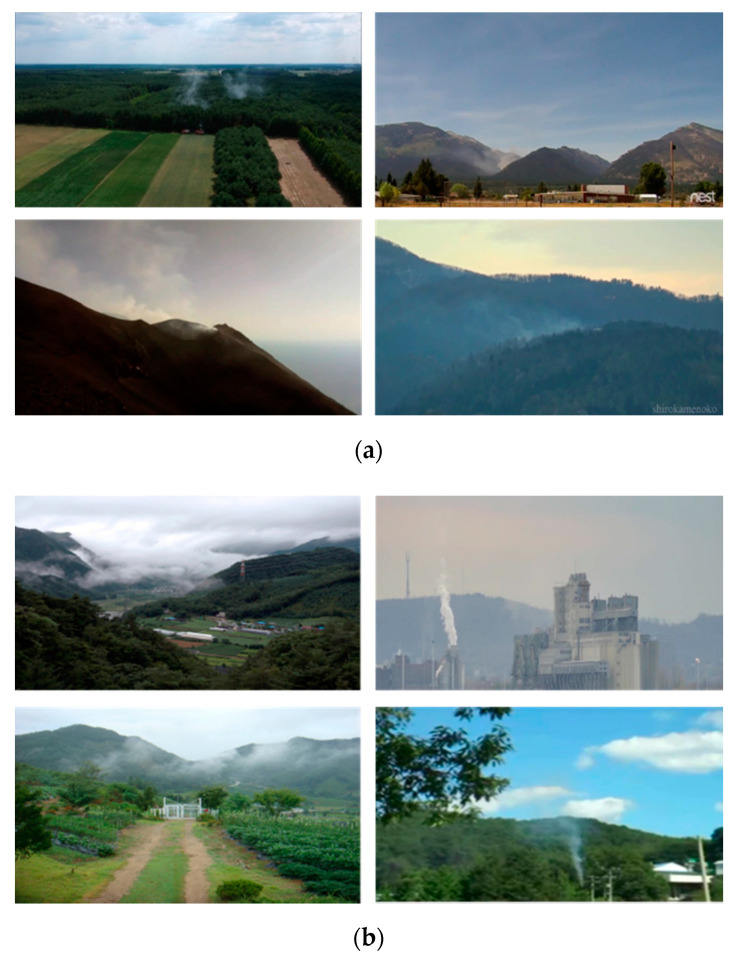
Examples of wildfire smoke and smoke-like cases: (**a**) An initial state of wildfire smoke captured from a camera installed on the surveillance tower of mountain and (**b**) various smoke-like cases such as clouds, fogs, and chimney smoke.

At the initial stage of wildfire occurrence, smoke occurs earlier than flames. Therefore, for the early extinguishing of a wildfire, it is necessary to effectively detect wildfire smoke that occurs during the early stages. However, early wildfire smoke is small in size and has similar properties as cloud, fog, and even chimney smoke, which have a visual appearance similar to wildfire smoke, as shown in Figure 1.

In this study, we focus on a proposed algorithm that can distinguish between actual wildfire smoke and smoke-like cases such as clouds, fog, and chimney smoke, during the daytime, through a real-time image analysis using deep learning methods. In particular, wildfire smoke spreads at different speeds depending on the wind, environmental conditions, circumstances, and distance from the camera. Therefore, instead of using a single image, we extract spatiotemporal information from sequential images to reflect various characteristics of wildfire smoke and ensure the accuracy of the wildfire detection.

The remainder of this paper is structured as follows. In Section 2, we present an overview of the related studies on wildfires based on a camera image. Section 3 provides the details of our proposed method in terms of the deep learning model. Section 5 provides a comprehensive evaluation of the proposed method through various experiments. Finally, some concluding remarks are given in Section 6.

## 2. Related Studies

To prevent damage from wildfires, studies on wildfire detection have been steadily conducted in the field of computer vision. Studies based on images compiled through such detection can be classified into two types: Hand-crafted feature and machine-learning based fire detection, and deep neural network (DNN) based fire detection. In this section, some representative algorithms used in two different approaches are introduced, and their advantages and disadvantages are analysed.

### 2.1. Hand-Craft Features and Machine Learning Based Wildfire Detection

In the case of wildfire detection based on handcrafted features and machine learning, the first step is to extract feature vectors based on user-specified features such as colour, motion, optical flow, and shape. The extracted feature vectors are applied to a rule-based decision, support vector machine (SVM), random forest, and neural network and are used for wildfire classification.

Reviewing some representative methods using handcrafted features, Chunyu et al. [15] used colour-based decision rules to determine the candidate regions of wildfire smoke. This method measures the motion of a fire using an optical flow in the determined candidate areas and determines whether it is a fire, according to the decision rule. Kim et al. [11] proposed wildfire smoke detection based on a colour model. In a fixed camera, the background of the image is extracted, and the region with a pixel change is designated as the region of interest (ROI). To determine whether a fire has occurred, K-temporal information on the colour shapes is generated from the extracted ROI. Töreyin et al. [16] proposed a method for identifying wildfire smoke based on background subtraction and temporal-spatial wavelet transformation. The spatial wavelet transformation at the edge of the moving area in the current frame was used to identify a reduction of the high-frequency components and determine whether it shows smoke. In a similar way, Gubbi et al. [17] analysed the characteristics of the image by applying wavelets and an SVM, demonstrating more reliable results.

In addition, Yuan et al. [18] detected wildfire smoke by applying an accumulative motion model rather than a single motion model in an integrated image to increase the accuracy through local binary patterns (LBPs) and a histogram of local binary pattern variance (LBPV) pyramid. With this method, LBP and LBPV histograms were extracted after decomposition into a 3-stage image pyramid. The extracted LBP and LBPV were connected by a single high-level feature vector, and neural network classifications were used to determine whether wildfire smoke was shown.

Chen et al. [19] extracted candidate pixels of wildfire smoke from areas that were moved based on the colour characteristics and then extracted the dynamic features to determine whether they showed smoke.

Ko et al. [20] extracted wildfire smoke candidate regions based on moving smoke-coloured objects. A histogram of gradient and that of oriented flow were extracted for the feature descriptors, and the dimensions of the feature vectors were then reduced by applying a bag-of-features to the extracted feature descriptors. For wildfire verification, this method creates a volume for the smoke region and applies the bag-of-feature features to a random forest classifier.

The handcrafted feature-based methods discussed thus far have a problem in that a programmer must find an optimal feature vector and classifier for classifying wildfire smoke, and the smoke detection performance is highly dependent on choosing an effective feature extraction and classifier. However, if the test data are changed (for example, from a fixed camera to a moveable camera), the existing feature vector may degrade the performance. Therefore, recent wildfire smoke detection methods mainly use a DNN, which is an end-to-end learning method that can apply processes, from the feature extraction to the classifier design, concurrently.

### 2.2. Deep Neural Network Based Wildfire Smoke Detection

In camera-based wildfire detection, many studies have increasingly attempted to detect wildfires using a convolutional neural network (CNN), which is a derivative algorithm of a DNN for image recognition. These CNN-based studies showed a better performance than existing handcraft feature and machine-learning based methods [21]. In this subsection, we review some representative studies on detecting wildfire smoke based on a CNN.

Zhang et al. [22] used AlexNet [23] as the backbone network for feature extraction and classification. This method additionally checks the location of the fire region when the entire image contains a wildfire. Yin et al. [24] proposed the use of deep normalisation and a CNN (DNCNN) for image smoke detection. This method replaces traditional convolutional layers with normalisation and convolutional layers to accelerate the training process and boost the performance of smoke detection. Aslan et al. [25] proposed a two-stage training method for deep convolutional generative adversarial neural networks (DC-GANs). This method trains the DC-GANs with real images and noise vectors, and the discriminator is separately trained using the smoke images without a generator. Before training the networks, the temporal evolution of smoke is also integrated with a motion-based transformation of images as a pre-processing step. Khan et al. [26] proposed an energy-efficient system based on a deep CNN for early smoke detection in both normal and foggy environments. This method takes advantage of the VGG-16 architecture [27], considering its sensible stability between accuracy and time efficiency. In addition, Xu et al. [28] proposed a deep saliency network that aims to highlight the important wildfire regions in an image. The pixel- and object-level salient CNNs are combined to extract the informative smoke saliency map. An end-to-end framework for salient smoke detection and prediction is proposed for application in video smoke detection.

Although the abovementioned CNN-based wildfire detectors showed a better performance than traditional handcrafted-based methods, the existing CNN models require many parameters and a large amount of computational time for training. In addition, the CNN-based methods discussed thus far generally detect wildfire smoke by applying a CNN to still images. However, as shown in Figure 1, because wildfire images are mainly taken from a long-distance camera, the moving speed is very slow, and in terms of visual appearance, there are similar characteristics to clouds and fog. As the distinguishing characteristic of wildfire smoke, it rises upwards and spreads over time. Therefore, to increase the detection accuracy of wildfire smoke, it is necessary to distinguish wildfire smoke from other smoke-like cases by grasping the motion and diffusion characteristics of the smoke in successive frames. To reflect the dynamic characteristics of wildfire smoke, wildfire detection methods based on CNNs and recurrent neural networks (RNNs) [29] that consider time series information have been proposed.

Lin et al. [30] proposed a joint wildfire detection framework based on a faster RCNN and a 3D CNN. A faster R-CNN [31] is first used to realise the smoke target location based on static spatial information. Then, the 3D CNN recognizes smoke by combining dynamic spatial–temporal information. However, 3D CNNs still generate feature maps based on a CNN, and thus there is a limit to reflecting the dynamic characteristics of smoke. An RNN [29], which can reflect time series information in deep learning, has a structure that combines the output information with the input of the next time zone and outputs it considering the past information. Filonenko et al. [32] proposed a combination of a CNN and an RNN to detect smoke in the spatial and temporal domains. The CNN part automatically generates low-level features, and the RNN part recognises smoke by finding the relationship between the features in different frames of the same event. However, an RNN has a vanishing gradient problem if the time between the occurrence of the currently extracted information and the previously extracted information is long.

To overcome this problem, long short-term memory (LSTM) [33] was proposed. The LSTM solves the vanishing gradient problem by adding the cell state during the hidden stage of the RNN. The LSTM solves the long-term dependency problem by considering not only the immediately prior information but also previous macroscopic data. Kim and Lee [34] also used a faster R-CNN to detect suspected regions of fire and non-fire based on their spatial features. After summarising the features within the bounding boxes in successive frames, LSTM is used to classify whether there is a fire within the short term. Decisions for short consecutive periods are combined by a majority vote for a final decision during a long-term period. However, in the case of the LSTM, the output approaches one direction as an input according to the chronological sequence, although this has a limitation in that it does not consider the surrounding timeframe [35]. However, in the case of LSTM, the output approaches one direction as input according to the chronological sequence. This means that we do not consider the around time.

Bi-LSTM [35] considering both directions was proposed to solve the problem of considering a unidirectional input. Bi-LSTM considers both directions, unlike the existing LSTM, by adding the reverse direction to the existing forward direction. Cao et al. [13] proposed the attention enhanced Bi-LSTM (ABi-LSTM) for video-based wildfire smoke recognition. The ABi-LSTM consists of a spatial feature extraction network, a Bi-LSTM, and a temporal attention sub-network. ABi-LSTM can not only capture discriminatory spatiotemporal features in image patch sequences, it also pays different degrees of attention to different patches to detect a wildfire. However, because this method only targets indoor near-field fire smoke, there is a limit to detecting distant wildfire smoke in natural environments.

A wildfire detection technique using a gated recurrent unit (GRU) [36,37], which has improved the recognition speed by simplifying the cells constituting the LSTM layer, has also recently been introduced. Karthy et al. [37] proposed LSTM- and GRU-based deep learning architectures for smoke prediction. Neither model uses only the outputs of the most recent layer for smoke detection, such as in other RNN models, but mainly uses knowledge of previously obtained outputs over a period of time.

RNN- or LSTM-based wildfire smoke detection methods using consecutive images generally show a better performance than methods based on still images. In particular, in the case of handcraft-based wildfire detectors, the detection performance is highly dependent on the feature descriptors, although in the case of a DNN-based wildfire detector, this dependency can be weakened. Therefore, in this study, to achieve a robust wildfire smoke detection system while minimising false detections even under various weather conditions and environments, the features are first extracted from candidate regions based on a CNN, and an LSTM is used for sequentially verifying the candidate regions. However, because an LSTM is a heavy structure requiring numerous computations, we propose a teacher–student model that can perform real-time computations by reducing the number of layers without reducing the accuracy of the LSTM.

### 2.3. Contributions of this Study

In this paper, we focus on the detection of early wildfire smoke, and try to detect highly reliable wildfire smoke through two processes: Wildfire candidate smoke detection using a CNN and verification using a lightweight shallow LSTM. The contributions of this study are as follows:

(1) The wildfire smoke input from the surveillance tower to the camera is characterised by an extremely slow spread. Therefore, it is an immensely difficult task to analyse the motion of the smoke for each frame. Instead of analysing the motion of the smoke in every frame, a frame with outstanding motion is declared as a keyframe. Next, the spatial-temporal feature of wildfire smoke was analysed by examining the previous N consecutive frames centred on the keyframe region. Because only the key frame is inspected, the time for smoke detection can be reduced, and even small movements of small or distant wildfires can be detected.
(2) To accurately determine wildfire smoke and non-wildfire smoke among candidate regions detected by the You-Only-Look-Once (YOLOv3) network [38], we additionally go through a verification process that considers the time series information. For this, we used the LSTM to determine the wildfire smoke based on a high reliability by considering the motion of the smoke for a certain period of time.
(3) Non-fire objects such as clouds, fog, and chimney smoke have an extremely similar visual appearance as wildfire smoke. Therefore, to classify them more effectively, it is necessary to construct a flexible classifier using a soft label rather than a hard label of the training data. In this study, an LSTM-based classifier is constructed based on the teacher–student framework. The teacher–student framework has the advantage of being able to flexibly configure existing classifiers using a soft label, and at the same time conduct a model weight reduction. We call the light-weighted LSTM model based on the teacher-student framework a shallow student LSTM.

**Figure 2:**
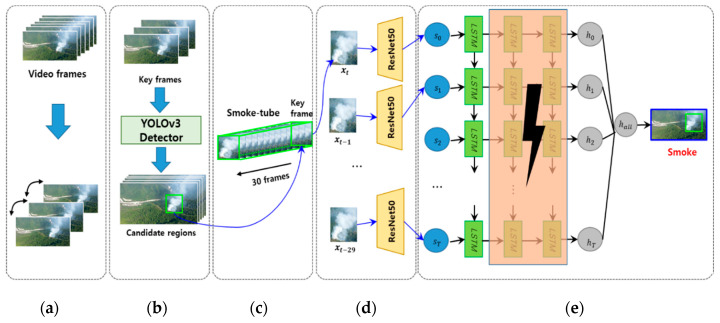
Overall procedure for detecting wildfire smoke: (**a**) Keyframe extraction, (**b**) smoke candidates detection, (**c**) smoke-tube configuration, (**d**) spatial feature extraction, and (**e**) verified wildfire smoke based on shallow student long short-term memory (LSTM); in this step smoke is detected as a student LSTM composed of only one layer obtained in the proposed teacher–student learning framework. The pink part in the figure shows the removed LSTM layers.

Figure 2 shows the overall procedure of the proposed method. First, we detect keyframes that reflect the slow motion of the smoke in the input image (Figure 2a). The wildfire smoke prediction algorithm is applied only for the key frames, and the wildfire smoke candidate regions are searched only from the key frames using the YOLOv3 detector (Figure 2b). To construct the time-series information for the detected region, a smoke-tube is formed by combining the previous 30 frames of the key frame (Figure 2c). The spatial features are extracted from each frame of the smoke tube and input into each corresponding LSTM (Figure 2d). The deep LSTM is reduced in weight by the teacher–student framework and converted into a shallow LSTM model, and the wildfire smoke is then verified based on the shallow LSTM (Figure 2e).

## 3. Candidate Wildfire Smoke Detection

### 3.1. Detection of Key Frames

Unlike smoke that occurs indoors, in the case of wildfire smoke, a CCD camera is installed at a high position to observe wildfires in a wide area, and thus even if actual wildfire smoke occurs, it is often too small to identify. In addition, because the focal length of the CCD camera is long, the smoke has a slower motion than in reality. Therefore, if a CNN or an LSTM is applied to each frame as in a conventional wildfire smoke detection method, the processing time is increased and a problem occurs in that the motion information of the smoke is given little consideration because of the slow motion characteristic. To solve this problem, in this study, a frame is selected as a key frame when it includes a partial region where severe motion changes occur rather than viewing the entire frame, as inspired by [39]. Subsequently, to verify whether smoke is included, a CNN and an LSTM are applied to the surrounding frames centring on the keyframes (Figure 2c).

The key frame checks whether there is a difference in motion between the previous key frame and the current frame. To this end, Gaussian smoothing is first applied to remove the noise of the frame, and the frame is divided into N × N patches. The difference in value is calculated for each patch between the previous key frame and the current frame, and if a movement of more than a certain threshold value occurs in one or more local patches, the current input frame is set as a key frame, as shown in Figure 1a. Otherwise, the next frame is input. This process is repeated until the next key frame is detected, and when the key frame is detected, post-processing for smoke detection is applied.

### 3.2. Detection of Candidate Smoke Regions Using YOLOv3

Unlike a conventional pixel-motion based method [39], we detect candidate smoke regions using a CNN-based object detector. The motion region between frames is not used as a candidate region because there are many types of motions such as clouds, fog movements, and tree shaking in surveillance camera images. In addition, a surveillance camera is installed at the top of the mountain, and strong winds may cause the camera to shake. In this case, the entire frame may be detected as a candidate region, resulting in erroneous wildfire determination. Conversely, when the distance between the wildfire and the surveillance camera is significant, the motion of the wildfire smoke in the image may appear to be extremely small, and thus such a wildfire smoke region may not be detected during the pre-processing based on a simple motion.

In this study, we find keyframes with motion during the pre-processing, and obtain smoke candidate regions from only keyframes using a CNN-based detector, and finally determine the presence of a wildfire through an LSTM-based post-processing. Therefore, the purpose of a CNN-based candidate region detector is to detect a sufficient number of candidate regions, including real smoke and smoke-like objects; minimise missing regions; and deliver them to the post-processing.

To construct a detector suitable for wildfire smoke, we compared and evaluated various recently proposed CNN-based object detectors, such as Faster-RCNN [31], RetinaNet [40], CornerNet [41], CenterNet [42], YOLOv3 [38], and ELASTIC-YOLOv3 [43]. We then compared two evaluation metrics, i.e., the processing speed and recall, for measuring the missing minimisation. As a result of the experiment, we determined that YOLOv3 achieves the best performance based on two evaluation items, and detects wildfire smoke candidate regions for different keyframes. The test results of various CNN-based smoke detectors are detailed in Section 4.

### 3.3. Construction of Smoke-Tube

**Figure 3:**
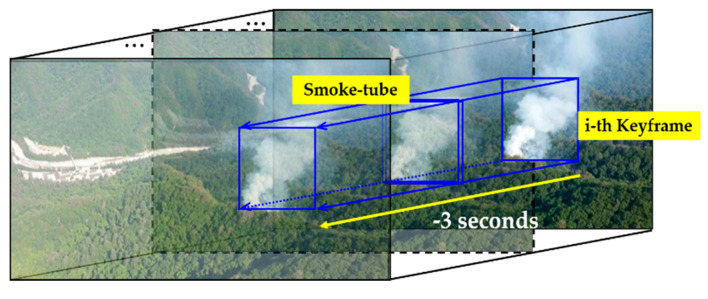
Smoke-tube configuration by considering the 30 frames for the previous 3 s based on the region detected in the current key frame.

In a video recorded by a surveillance camera, there are various types of clouds and fog that have an extremely similar appearance as the smoke from a wildfire. Therefore, to distinguish these from actual smoke, it is necessary to consider the temporal movement of the smoke, which changes by the wind or ignition material. To this end, in this study, the smoke candidate region of the keyframe is first detected using YOLOv3, and a smoke-tube is then constructed using frames of the previous 3 s at the same position as the corresponding candidate region which is similar number of frames suggested by [13,20,39], as shown in Figure 3. Based on the smoke-tube, the wildfire smoke region is verified through a post-processing when considering the spatiotemporal features.

When constructing a smoke-tube, if we collect all frames during the previous 3 s and use them for the LSTM, the LSTM performance can be degraded because the motion is too fine owing to the slow diffusion speed of smoke. Therefore, to shorten the processing time and apply an effective smoke verification, we extract only 10 frames per second (fps) instead of using all frames for the smoke-tube. In [13], 5 fps were extracted for a total 4 s, but because the fine motion of the smoke could be missed, in this study, a total of 30 frames, at 10 fps, are stored in the smoke-tube for a total of 3 s in consideration of the processing time and memory usage. After the smoke-tube is constructed, all frames in the smoke tube are normalised to a size of 216 × 216 which is the input size of ResNet50 for feature extraction.

## 4. Wildfire Smoke Verification Based on a Student Shallow LSTM

### 4.1. Spatial Feature Extraction of Smoke-Tube

We use the LSTM to consider the time-series characteristics of smoke tubes composed of spatial feature vectors for final smoke verification. Each frame of the smoke-tube is input into the corresponding input unit of the LSTM, although the frame itself has large dimensions of a feature vector. Therefore, through an additional step, the optimum feature value is extracted from each frame of the smoke tube and applied to the input unit of the LSTM.

**Figure 4:**
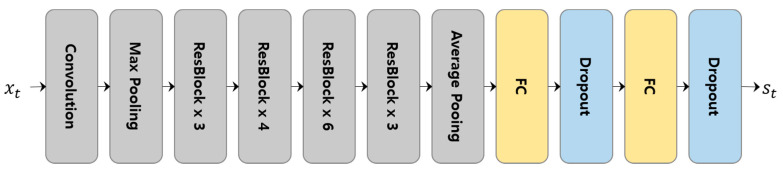
Modified ResNet50 structure for extracting smoke features. Here, $x_{t}$ represents the *t*th input region and $s_{t}$ represents the 1024-dimensional output feature vector for the region.

To extract more valid features from the images in the smoke-tube, we apply fine-tuned ResNet50 [44] to each region of the smoke-tube and reconstruct the smoke-tube into a set of features that are robust to smoke verification. The ResNet50 model was pre-trained using the ImageNet dataset. As shown in Figure 4, we reconstruct the ResNet50 model by adding two fully connected layers (FC) instead of the top-level classifier for classification and fine-tuned it using wildfire smoke data. Two fully connected layers having outputs of 2048 and 1024 dimensions, respectively, and a dropout layer are placed behind each FC layer to minimise an over-fitting. Therefore, each image of a smoke-tube is represented as a 1024-dimensional feature vector.

### 4.2. Fire Verification Using Deep LSTM

Several sequential neural network (SNN) algorithms that can analyse the time-series information have recently been introduced. For example, LSTM [33], Bi-LSTM [35], and a gated recurrent unit (GRU) [36] were introduced. Bi-LSTM [35] is an algorithm developed to solve the shortcomings in existing LSTMs that only considers the forward connection and not the backward connection, and thus future data cannot be used for inference. GRU [36] reduces the computation of updating the hidden state while maintaining a solution to the long-term dependency problem of the LSTM. In other words, the GRU simplifies the structure of the complex LSTM, which is similar in performance to the LSTM. However, because wildfire smoke has a distinct characteristic of spreading over time, we only need to consider the forward connection in the LSTM. In addition, in the performance comparison experiment (see Section 5), because the basic LSTM showed a better performance than the reduced GRU, we verified the wildfire based on the forward LSTM with the output of the smoke-tube and ResNet50.

**Figure 5:**
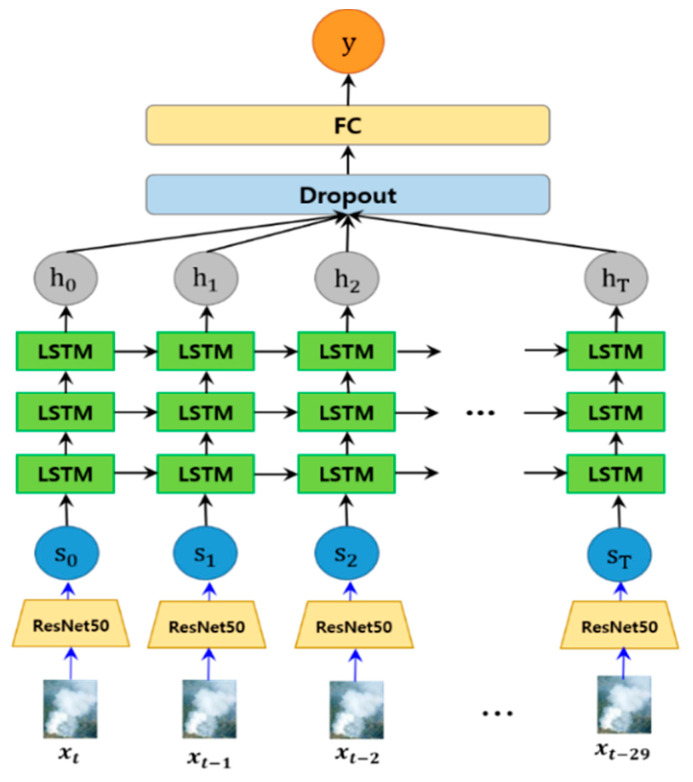
Structure of LSTM consisting of three layers. 30 frames of smoke candidates are applied to the input unit continuously through ResNet50.

As shown in Figure 5, the smoke tube for the previous 30 frames is constructed around the smoke candidate area of the keyframe. ResNet50 is applied to each frame of the smoke tube to construct a spatial feature vector, which is sequentially input into the LSTM model. After concatenating the LSTM outputs into one feature vector for each sequential information, the final probability values for wildfire smoke and non-smoke are derived through the dropout layer and the fully connected layer.

The deeper LSTM model performs well in large-scale continuous data recognition owing to its learning ability. However, as the number of layers increases, there is a disadvantage in that training becomes difficult, and the amount of memory and test time increase owing to numerous parameters [45]. Therefore, for real-time wildfire smoke detection, an additional algorithm is needed to lighten the deep LSTM model while maintaining the classification performance.

### 4.3. Teacher–Student Framework for Constructing Shallow LSTM

In this study, a teacher–student framework [46] is used to develop a shallow LSTM model through a weight reduction while maintaining the smoke classification performance of the deep LSTM model. The teacher–student framework constructs a deep and wide teacher model with a high performance based on a large amount of training data and deep layers, and constructs a shallower student model with an equal performance based on the teacher model [47]. Through this process, the shallow LSTM has the advantage of reducing the weight of the model while maintaining the performance of the existing teacher model.

**Figure 6:**
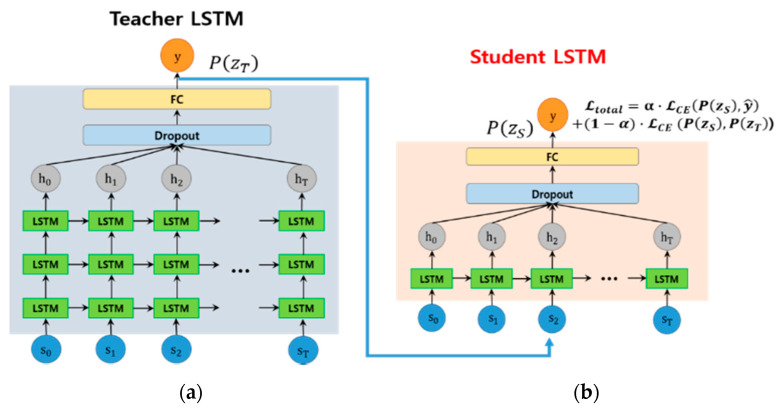
The proposed teacher–student framework for constructing a shallow student LSTM, (**a**) a teacher LSTM consists of three layers and (**b**) a shallow student LSTM consists of a single layer.

Figure 6 shows the teacher–student framework proposed in this paper. As shown in Figure 6a, a deep LSTM (teacher) composed of three layers is reduced to a shallow LSTM (student) model composed of one layer (Figure 6b) through a learning process.

The training smoke dataset is divided into dataset A for teacher learning and a larger dataset B for student learning. To train the teacher LSTM model, training set A is provided as the basis for the training component.

(1) $$A=\left\{\left(x_{i},y_{i}\right)|i=1,2,\ldots N\right\},$$

where $x_{i}=\left(x_{i1},x_{i2},\ldots ,x_{iM}\right)$ is an input vector with *M* (1024) dimensions and $y_{i}=\left\{g_{1},g_{2},\ldots ,g_{C}\right\}$ is a scalar (*C* is the number of classes and has two classes), representing the class marked by the expert. Dataset A, labelled with a scalar 1 (smoke)/0 (non-smoke), is called a “hard label”. The teacher LSTM is then trained to minimise the classification error using labelled training set A.

The trained dataset B is then input to the teacher LSTM, which is trained using the corresponding hard labels. Unlike those of training dataset A, each sample of dataset B is applied to the teacher LSTM to calculate the class probability vector according to the results of Equation (3), and relabel the original dataset B. After all samples included in dataset B have been trained, a new dataset $B^{*}$ is constructed as follows:

(2) $$B^{*}=\left\{\left(x_{i}^{*},\;p_{i}^{*},{\hat{y}}_{i}\right)|i=1,2,\ldots N^{*}\right\}.$$

The new dataset $B^{*}$ is transcribed with a class probability $p_{i}^{*}$, which is called a “soft label” as opposed to a hard label, and hard class label $\hat{y}$ is marked by an expert [47].

To compose a high-performance teacher LSTM, deep LSTMs composed of three layers inspired by [48], with each LSTM module consisting of 128 cells, as shown in Figure 5, were applied. To determine the number of cells with optimal performance in LSTM, the number of cells per layer was increased by multiples from 32 to 1024 using Cao et al. [13]’s method. As a result, when using 32 cells per layer, the F1-score showed 84.74% performance, and then continued to increase, showing the best performance at 87.85% at 128 cells. However, when more than 128 cells were used, the F1-score gradually decreased again. In particular, when using 1024 cells, the performance decreased to 82.87%.

The teacher LSTM must deliver its own learning ability properly in the student LSTM, and this ability can be delivered through the probability value for each class estimated from the teacher LSTM. However, some of the class output probabilities of the teacher LSTM are close to zero, and thus the information might not be delivered properly during the backpropagation learning. Therefore, to soften the probability value for each class, temperature *T* is added to the existing softmax as follows [49]:

(3) $$P\left(z_{i}\right)=\frac{exp\left(\frac{z_{i}}{T}\right)}{\sum_{j}exp\left(\frac{z_{j}}{T}\right)},$$

where $z_{j}$ is the logit value. If *T* = 1, we obtain the standard softmax function. As *T* increases, the probability distribution produced by the softmax function becomes smoother, giving more information about the class that the teacher found to be more similar to the predicted class. In this study, *T* = 2 was set by referring to [49].

To make the student LSTM lighter than the teacher LSTM, we used one LSTM layer, and the number of cells in each LSTM module was 24. Student LSTM training uses a B* dataset composed of soft labels, and the loss function used for model training is as follows.

(4) $${ℒ}_{total}=\alpha \cdot {ℒ}_{CE}\left(P\left(z_{S}\right),\;\hat{y}\right)+\left(1-\alpha \right)\cdot {ℒ}_{CE}\left(P\left(z_{S}\right),\;P\left(z_{T}\right)\right),$$

where ${ℒ}_{CE}$ represents the cross entropy loss, and $P\left(z_{S}\right)$ and $P\left(z_{T}\right)$ represent the output (probability) of each student LSTM and teacher LSTM. The first term in Equation (4) represents the cross-entropy between the hard class labels $\hat{y}$ and the output $P\left(z_{S}\right)$ of the student LSTM, and the second term is the cross-entropy between the output $P\left(z_{S}\right)$ of the student LSTM and the output $P\left(z_{T}\right)$ of the teacher LSTM. Parameter α is an adjustable value that maintains the balance between the two terms and is set to 0.5 in this study. Through the teacher–student learning process using Equation (4), the student LSTM applies fewer parameters than the teacher LSTM, but achieves almost the same learning performance.

During the test process, candidate smoke-tube inputs are applied to the trained student LSTM to be verified as wildfire smoke and non-smoke.

## 5. Experimental Results

In this section, to prove the effectiveness of the proposed method, we measure the objective performance through several comparative experiments. To this end, we compare the performance of wildfire smoke detection with various comparison algorithms and the proposed method. In addition, by comparing the performance and model size of teacher LSTM and student LSTM, we prove that the proposed student LSTM model maintains the performance while reducing the size.

### 5.1. Dataset

**Figure 7:**
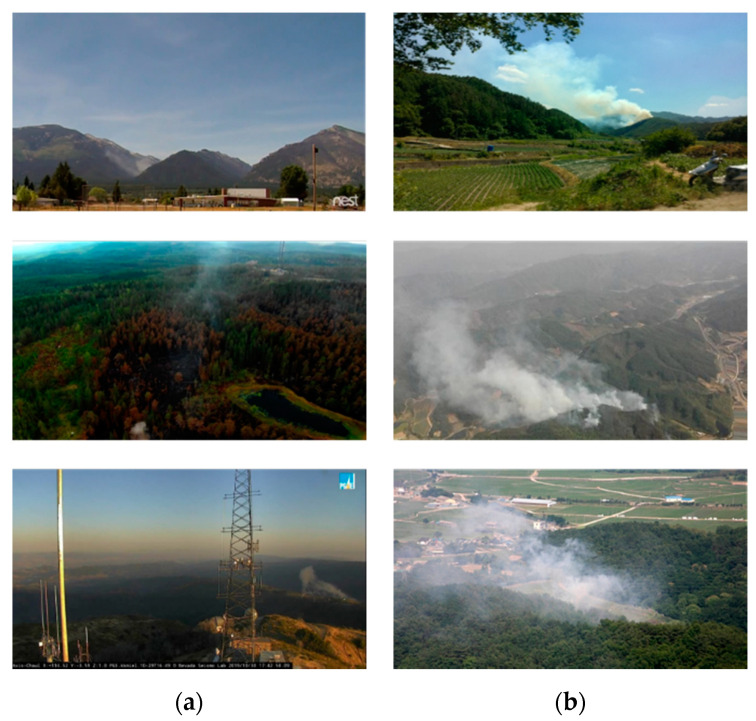

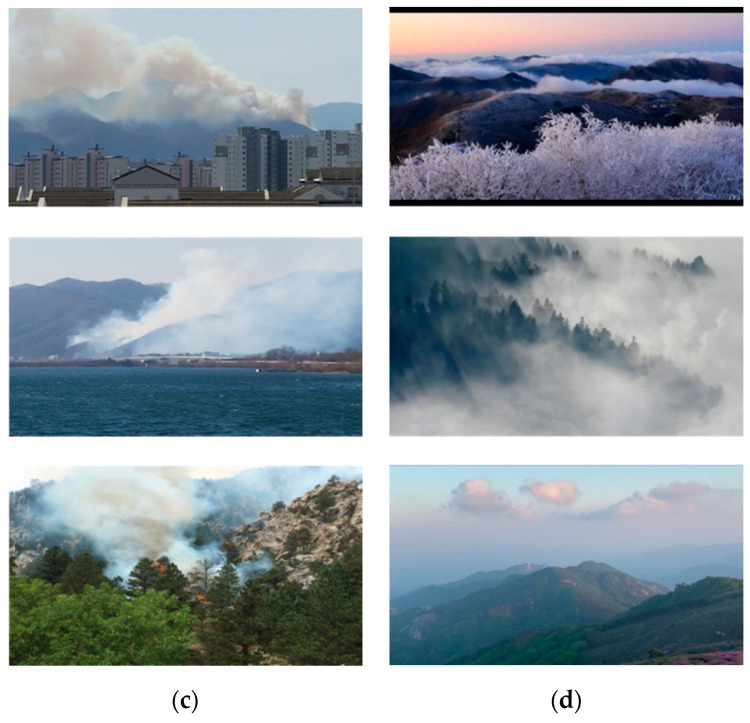
Samples of various benchmark wildfire smoke and smoke-like sequences used in training and testing: (**a**) Small-, (**b**) medium-, and (**c**) large-sized wildfires, and (**d**) cloud and fog.

We used a dataset consisting of still images for candidate smoke detector training and a dataset consisting of video sequences for LSTM verifier training. In addition, to evaluate the performance of each module, separate test video sequences were collected. Currently, there are very few open benchmark datasets [50,51] in the field of fire and smoke detection research, and the quality of the images is extremely poor because datasets have been published for many years. However, because there were many problems in using the benchmark dataset, we needed to construct a new dataset. The currently used CCTV approach uses an excellent image quality of normal and full HD, with a frame rate of 20 fps or higher. Therefore, to compose a new benchmark dataset, we collected various wildfire smoke data by combining wildfire videos recorded directly and downloaded from YouTube. Camera images may be subject to colour interference by sunlight, but cameras installed for wildfire detection are mainly facing downwards, so this is very rare. The case of light reflection due to sunrising or sunset in the early morning or evening was excluded. Figure 7 shows samples of the benchmark wildfire sequences used in this study. We divided the dataset into large, medium, and small sizes according to the amount of smoke proportional to the image size.

The dataset for the candidate smoke detector training consisted of only 2022 smoke classes excluding non-smoke. This is because smoke and smoke-like objects have little difference in still images, and the detector aims to detect only smoke regions without missing them. The dataset consists of images of various resolutions and contains wildfire smoke of various sizes, from large wildfire smoke to small wildfire smoke.

The dataset for the LSTM verifier included wildfire smoke videos as well as non-wildfire smoke videos rather than fire-flames for testing of early wildfire detection. The wildfire smoke videos consisted of videos captured in a variety of environments, from a small initial smoke to large spreading smoke, smoke captured from a large distance, and smoke captured from a short distance. In the case of non-wildfire smoke videos, the factory chimney, fog, and clouds are included. The video dataset consisted of images with a resolution of 640 × 480 or higher at a frame rate of 25 fps. There were a total of 70 wildfire videos, of which 12 were used for testing. A total of 64 non-wildfire videos were used, 12 of which were also used for testing. The training data was selected one by one in the training process so that wildfire and non-smoke data having various shapes, movements, seasons were included. In particular, because wildfires are mainly concentrated in spring, autumn, and winter, the three-season videos were included in the training and test data. The wildfire smoke test data was selected to include the following conditions: (1) The size of wildfire smoke must be different, (2) the location of wildfire smoke must be different, (3) the direction or shape of wildfire smoke should be different, (4) the three seasons of wildfire should be distributed. Likewise, the test data for non-smoke were selected to include the following conditions: (1) Data including clouds, fog, chimney smoke, etc. should be included, (2) the size of the non-smoke should be different, (3) the location of the non-smoke should be different, and (4) the three seasons of the non-smoke should be distributed. To ensure the objectivity of the experiment, the test data of wildfire smoke and non-smoke were selected to satisfy these conditions as much as possible.

Table 1 describes the resolution, number of frames, fps, and seasons for the videos from the test dataset.

### 5.2. Implementation Details

The experiments were conducted using an Intel Core i7-7700K CPU (Intel, Santa Clara, CA, USA) processor and an NVIDIA GeForce GTX 1080 Ti GPU (NVIDIA, Santa Clara, CA, USA) running Microsoft Windows 10 (Microsoft, Redmond, WA, USA). The proposed method was implemented on a Keras with a TensorFlow backend.

The training of the YOLOv3 detector used in the proposed method was fine-tuned using the weights pre-trained with ImageNet. In addition, YOLOv3 was changed to nine anchors in consideration of the shape of wildfire smoke. YOLOv3 training was conducted using a learning rate of 0.002, batch size of 64, and 100 epochs based on an input image of 416 × 416 × 3 (channel). The smoke-tube was extracted around the smoke candidate area from a total of 30 frames generated for 3 s at 10 fps. The smoke-tube was fed into ResNet50 + LSTM. ResNet50 used here uses weights pre-trained with ImageNet beforehand, and is fine-tuned by adding two fully connected layers with a dropout of 0.5 and the ReLu activation function.

The smoke tube consists of 30 images of 216 × 216 × 3 (channel), and by inputting each sequence into the fine-tuned ResNet50, a feature vector of 1024 (feature vector) × 30 (sequences) is obtained. The extracted feature vector is input into the LSTM, and finally, the probability values for smoke and non-smoke classes are generated. To design a shallow student LSTM, we first need to define a teacher LSTM model. We designed a three-layer LSTM based on 128 cells as a teacher LSTM. The student LSTM was designed as a one-layer LSTM based on 24 cells. To learn the two LSTMs, an Adam optimiser was used with a learning rate of 0.001, a batch size of 128, and 500 epochs.

### 5.3. Performance Measurements

To evaluate the detection performance of wildfire smoke, this study used the precision, recall, and F1-score (which is the harmonic mean of the precision) based on the number of true positives (TPs), true negatives (TNs), false positives (FPs), and false negatives (FNs), which are widely used in two-class object detection.

(5) $$\mathrm{Precision}=\frac{\mathrm{TP}}{\mathrm{TP}+\mathrm{FP}}$$

(6) $$\mathrm{Recall}=\frac{\mathrm{TP}}{\mathrm{TP}+\mathrm{FN}}$$

(7) $$F1-\mathrm{score}=2\times \frac{1}{\frac{1}{\mathrm{Precision}}+\frac{1}{\mathrm{Recall}}}=2\times \frac{\mathrm{Precision}\times \mathrm{Recall}}{\mathrm{Precision}+\mathrm{Recall}}$$

In addition, to evaluate how well the wildfire smoke and non-smoke were detected in sequence units, the true positive rate (TPR) and true negative rate (TNR) were also measured as follows.

(8) $$\mathrm{TPR}=\frac{\mathrm{TP}}{\mathrm{TP}+\mathrm{FN}}$$

(9) $$\mathrm{TNR}=\;\frac{\mathrm{TN}}{\mathrm{TN}+\mathrm{FP}}$$

### 5.4. Performance Evaluation the Smoke Detectors

We first conducted comparative experiments using CNN-based state-of-the-art detectors to find the optimal smoke detector as a pre-processing step. Because the wildfire detection system must detect smoke in real time, its performance as well as speed are important factors for consideration. Therefore, we measured the accuracy of the one-stage detector YOLOv3 [38] and recently proposed a Faster R-CNN [31], RetinaNet [40], CornerNet [41], CenterNet-lite [42], and ELASTIC-YOLOv3 [43] object detectors. Experiments were run frame by frame on 24 wildfire smoke and non-smoke test sequences.

Table 2 shows the results of the comparative performance evaluation of the latest CNN-based object detectors. In the performance evaluation of Table 2, it is more important that FN is lower than that of FP. The FP can be removed in post-processing that considers motion afterwards, but the case of missing cannot be newly detected in post-processing. The most important role of the pre-processing smoke detection step is to reduce FP without missing candidate regions as much as possible. The model with the best F1-score performance, which shows the balance between the precision and recall, was ELASTIC-YOLOv3 at 68.72%. However, this method confirms that the FN and recall decreases considerably. In the case of a wildfire, this method is unsuitable because it is important to detect all existing wildfires. The model with the highest recall score is Faster R-CNN at 98.15%. However, Faster R-CNN has a processing speed of 0.255 s, which is unsuitable for real-time systems. The other three systems show a similar performance but have a drawback in that takes a high processing speed overall. Therefore, we adopted YOLOv3, which has the fastest processing time and high recall rate, as a wildfire smoke detector, although the F1-score is 4.5% lower than that of ELASTIC-YOLOv3.

### 5.5. Teacher–Student Model Selection

In this sub-session, we conducted several comparison experiments to construct a network that can consider the time information with a good performance. As described in related studies, various networks such as LSTM [34], Bi-LSTM [13], and GRU [37] have been used to consider the time-series information of the wildfire. During this experiment, because the verification of each algorithm was tested, the common input was used as the output of ResNet50. First, we measured and compared the wildfire verification performance of the LSTM [34], Bi-LSTM [13], and GRU [37] models to find an optimal teacher model. The candidate teacher model used in the experiment consisted of three layers, and the number of cells in each module was unified as 128. Table 3 shows the performance comparison of the three candidate teacher models using four metrics.

As shown in Table 3, the deep LSTM, which used three LSTM layers based on the F1-score, achieved the best performance at 87.85%. In addition, deep LSTM showed the best performance in the remaining three metrics, precision, recall, and TPR except TNR. Although the TNR is slightly inferior to other methods, the student model trained with a soft label has better TNR performance than the teacher model as shown in Table 4. In general, it is known that Bi-LSTM shows better results than LSTM in natural language processing, but it is judged that it is unnecessary to inspect feature patterns in both directions in patterns that diffuse over time, such as smoke. Therefore, we adopted a deep LSTM model composed of three LSTM layers as a teacher model.

After the teacher LSTM is constructed, training dataset B is then input into the teacher deep LSTM, which produces the class probability vector, and a new soft labelled dataset $B^{*}$ is constructed. To select the shallow student model, we trained shallow RNN-based candidate models (one-layer LSTM, Bi-LSTM, and GRU) with a soft labelled dataset $B^{*}$. The student models use one layer to compose a relatively small size compared to the teacher model, and the number of cells in the module was unified to 24. After training, we measured the performance of three RNN-based shallow models using the test dataset and showed the performance results in Table 4. As shown in Table 4, the performance of the student model for the LSTM, Bi-LSTM, and GRU on test datasets was 87.39%, 84.41%, and 84.48%, respectively. Among them, the LSTM-based student model achieved the best F1-score, showing only a 0.53% difference in performance from the teacher model. From the experimental results, we can see that the student model inherits the classification ability of the teacher model well.

**Figure 8:**
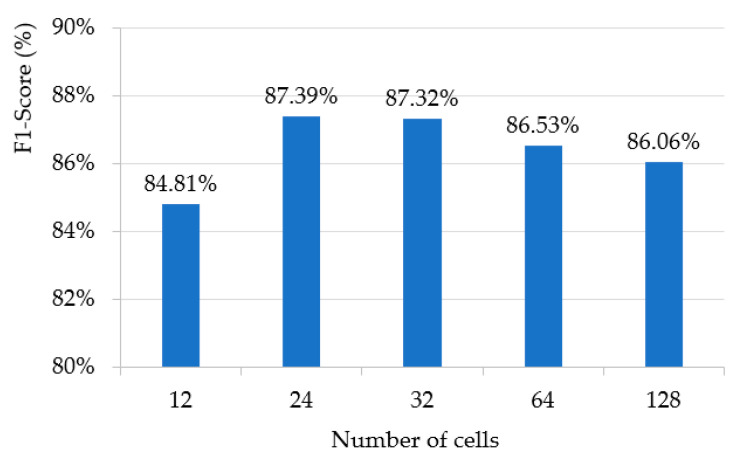
F1-score performance evaluation according to the number of cells of one LSTM for student model.

In Table 4, we used 24 cells per module for the student LSTM. Because the number of cells used in LSTM is closely related to the accuracy and processing time of the model, we measured the F1-score of wildfire smoke verification by changing the number of cells per module of one LSTM, which showed the best performance among the three RNN-based student methods. As shown in Figure 8, the result of one LSTM according to the number of cells has the best performance when using 24 cells, and it can be seen that the performance gradually decreases as the number of cells increases in 24 cells. This means that the performance does not improve as the number of cells increases. Rather, the use of a large number of cells indicates that learning may be hindered by storing values that are not good for predicting the results. Therefore, it was confirmed through an experiment that the 24 cells used in the student model were the optimal number, and this number helped further lighten the student model.

As a third experiment, we compared the number of parameters and processing time between the teacher model and the student model to test whether the student model actually became lighter and faster while maintaining the performance. As shown in Table 5, in the case of the student LSTM, only one layer was used and the number of cells in the layer was 24, which was 5-times less than that of the teacher, although the F1-score was only decreased by approximately 0.46%. By contrast, the number of parameters decreased by 8.4-times that of the teacher LSTM, and the processing time per frame also decreased by 0.019 s. From the experiment, we can observe that the student LSTM using only one layer maintains the performance of the wildfire smoke verification while reducing the amount of memory for parameter storage and processing time, enabling real-time wildfire fire detection.

**Figure 9:**
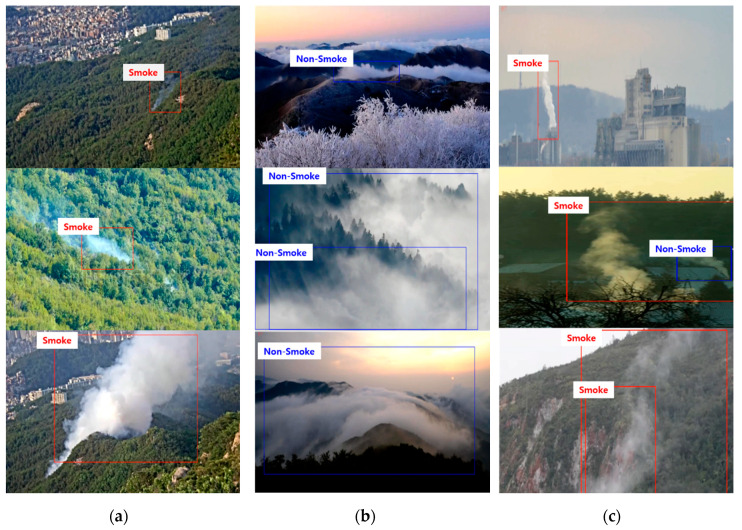
Wildfire smoke detection results using the proposed method: (**a**) Correctly detected smoke regions regardless of size, (**b**) correctly detected non-smoke regions such as clouds and fog, and (**c**) falsely detected smoke regions for chimney smoke and fog against a mountain background.

Figure 9 shows examples of the results of applying the test dataset to the proposed system based on a student LSTM with YOLOv3. As shown in Figure 9a,b, wildfire smoke and general clouds or fog are well distinguished in many frames, although as shown in Figure 9c, there are some cases in which the chimney smoke or fog is incorrectly classified. Therefore, for more accurate detection of wildfire smoke, it is necessary to improve an algorithm that can finely distinguish chimney smoke and fog in the post-processing stage as well as pre-processing.

## 6. Conclusions

In this study, we proposed a YOLOv3 and lightweight LSTM-based wildfire smoke detector using a teacher–student framework. The proposed method reduces unnecessary operations of the smoke detection system by detecting keyframes that contain significant motion instead of the entire frame. In addition, by applying YOLOv3 to the key frame only, the pre-processing time for detecting the candidate smoke region and the smoke-tube generation for a time-series analysis were reduced. In the performance evaluation of Table 2, it is more important that FN is lower than that of FP. The FP can be removed in post-processing that considers motion afterwards, but the case of missing cannot be newly detected in post-processing. The most important role of the pre-processing smoke detection step is to reduce FP without missing candidate regions as much as possible. To verify the wildfire smoke, we developed a student LSTM model that can reduce the number of model parameters and improve the processing time while maintaining a performance similar to that of the original deep LSTM by applying the teacher–student framework. Moreover, we proved the superiority of the proposed method for wildfire smoke detection through comparative experiments.

However, the current system still has a difficulty in distinguishing between chimney smoke or clouds that are similar in motion to wildfire smoke. Therefore, in future research, first, we plan to solve this verification error by upgrading the smoke-tube generation method and LSTM structure. Second, because it is important not to miss the smoke area in the pre-processing stage for wildfire smoke detection, it is necessary to newly construct a smoke detection model so that false negatives for the smoke area do not occur in the pre-processing stage. Third, we plan to continue research on a light weighting method for further simplifying the LSTM structure such that it is more suitable for real-time systems.

## Figures and Tables

**Table 1:** Detailed video configuration of the test dataset ^1^.

Type	Name	Resolution	Number of Frames	fps	Description	Season
Smoke	smoke01	1920 × 1080	806	25	Large size wildfire smoke	Winter
	smoke02	1920 × 1080	650	25	Small size wildfire smoke	Spring
	smoke03	1920 × 1080	232	25	Medium size wildfire smoke	Autumn
	smoke04	1920 × 1080	534	25	Small size wildfire smoke	Spring
	smoke05	1920 × 1080	985	25	Small size wildfire smoke	Spring
	smoke06	1920 × 1080	751	25	Large size wildfire smoke	Winter
	smoke07	1920 × 1080	795	25	Small size wildfire smoke	Spring
	smoke08	1920 × 1080	1490	25	Large size wildfire smoke	Spring
	smoke09	1920 × 1080	609	25	Small size wildfire smoke	Spring
	smoke10	1920 × 1080	677	25	Large size wildfire smoke	Spring
	smoke11	1920 × 1080	469	25	Small size wildfire smoke	Spring
	smoke12	1920 × 1080	305	25	Small size wildfire smoke	Spring
Non- smoke	chimney01	1920 × 1080	624	25	Small size chimney smoke	Spring
	industrial01	1920 × 1080	1401	25	Medium size industrial chimney smoke	Spring
	cloude01	1920 × 1080	204	25	Large size cloud	Winter
	cloude02	1920 × 1080	451	25	Medium size cloud	Autumn
	cloude03	1920 × 1080	533	25	Large size cloud	Spring
	cloude04	1920 × 1080	418	25	Large size cloud	Spring
	cloude05	1920 × 1080	266	25	Large size cloud	Winter
	cloude06	1920 × 1080	551	25	Large size cloud	Autumn
	cloude07	1920 × 1080	785	25	Medium size cloud	Spring
	fog01	1920 × 1080	501	25	Medium size fog	Winter
	fog02	1920 × 1080	684	25	Large size fog	Spring
	fog03	1920 × 1080	1444	25	Large size fog	Spring
Average		1920 × 1080	674	25		

^1^ The experimental test dataset can be provided upon request by email.

**Table 2:** Comparison of state-of-the-art methods for the object detection: YOLOv3, ELASTIC-YOLOv3, Faster R-CNN, RetinaNet, ConrnerNet-lite, and CenterNet.

Method	F1-Score (%)↑	Precision (%)↑	Recall (%)↑	TP (%)↑	FP (%)↓	FN (%)↓	Processing Time (s)
YOLOv3 [38]	64.17	48.32	95.51	47.24	50.53	2.22	0.014
ELASTIC-YOLOv3 [43]	68.72	54.91	91.82	52.35	42.98	4.66	0.017
Faster R-CNN [31]	52.89	36.2	98.15	35.95	63.36	0.67	0.408
RetinaNet [40]	48.87	32.79	95.88	32.33	66.27	1.38	0.081
CornerNet-lite [41]	39.92	25.15	96.67	24.93	74.2	0.85	0.316
CenterNet [42]	52.52	36.47	93.83	35.61	62.03	2.34	0.787

**Table 3:** Performance comparison of LSTM, Bi-LSTM, and gated recurrent unit (GRU) for finding an optimal teacher model. Deep LSTM model that includes three LSTM layers ^1^.

Models	F1-Score (%)↑	Precision (%)↑	Recall (%)↑	TPR (%)↑	TNR (%)↑
ResNet50 + deep LSTM [34]	87.85	86.06	89.72	89.72	80.02
ResNet50 + deep Bi-LSTM [13]	86.28	88.40	84.25	84.25	84.81
ResNet50 + deep GRU [37]	87.08	87.43	86.72	86.72	82.87

^1^ Because the source code of each algorithm for comparison is not open source, each method was implemented and tested similarly to each comparison paper.

**Table 4:** Performance comparison of shallow LSTM, Bi-LSTM, and GRU for optimal student model construction of the proposed method.

Teacher/Student	Label Type	Model	F1-Score (%)↑	Precision (%)↑	Recall (%)↑	TPR (%)↑	TNR (%)↑
Teacher	Hard	Deep LSTMs	87.85	86.06	89.72	89.72	80.02
Student	Soft	One LSTM	87.39	87.81	87.78	87.78	82.00
Student	Soft	One Bi-LSTM	84.41	84.79	84.03	84.03	79.29
Student	Soft	One GRU	84.48	84.89	84.07	84.07	79.44

**Table 5:** Comparison of parameters and processing time according to teacher–student model configuration. In the experiment, the parameters and processing time for ResNet50 were excluded.

Model	Number of Cells	F1-Score (%)	Number of Parameters	Processing Time (s)
Deep LSTM (teacher)	128	87.85	861,186	0.174
One LSTM (student)	24	87.39	102,146	0.155
